# Circulating Cell-Free DNA in Dogs with Mammary Tumors: Short and Long Fragments and Integrity Index

**DOI:** 10.1371/journal.pone.0169454

**Published:** 2017-01-12

**Authors:** Giorgia Beffagna, Alessandro Sammarco, Chiara Bedin, Chiara Romualdi, Marta Mainenti, Antonio Mollo, Laura Cavicchioli, Silvia Ferro, Davide Trez, Raffaella De Maria, Donato Nitti, Andrea Saccani, Michelangelo Campanella, Marco Agostini, Valentina Zappulli

**Affiliations:** 1 Department of Comparative Biomedicine and Food Science, University of Padua, Legnaro, Padua, Italy; 2 Department of Surgical, Oncological and Gastroenterological Sciences, University of Padua, Padua, Italy; 3 Istituto di Ricerca Pediatrica – Città della Speranza, Padua, Italy; 4 Department of Biology, University of Padua, Padua, Italy; 5 Department of Animal Medicine, Productions and Health, University of Padua, Legnaro, Padua, Italy; 6 Department of Veterinary Sciences, University of Turin, Grugliasco, Turin, Italy; 7 EuroClone, Pero, Milan, Italy; 8 Department of Comparative Biomedical Sciences, The Royal Veterinary College, London, United Kingdom; 9 UCL Consortium for Mitochondrial Research, London, United Kingdom; Cornell University, UNITED STATES

## Abstract

Circulating cell-free DNA (cfDNA) has been considered an interesting diagnostic/prognostic plasma biomarker in tumor-bearing subjects. In cancer patients, cfDNA can hypothetically derive from tumor necrosis/apoptosis, lysed circulating cells, and some yet unrevealed mechanisms of active release. This study aimed to preliminarily analyze cfDNA in dogs with canine mammary tumors (CMTs). Forty-four neoplastic, 17 non-neoplastic disease-bearing, and 15 healthy dogs were recruited. Necrosis and apoptosis were also assessed as potential source of cfDNA on 78 CMTs diagnosed from the 44 dogs. The cfDNA fragments and integrity index significantly differentiated neoplastic versus non-neoplastic dogs (P<0.05), and allowed the distinction between benign and malignant lesions (P<0.05). Even if without statistical significance, the amount of cfDNA was also affected by tumor necrosis and correlated with tumor size and apoptotic markers expression. A significant (P<0.01) increase of Bcl-2 in malignant tumors was observed, and in metastatic CMTs the evasion of apoptosis was also suggested. This study, therefore, provides evidence that cfDNA could be a diagnostic marker in dogs carrying mammary nodules suggesting that its potential application in early diagnostic procedures should be further investigated.

## Introduction

It is expected that human annual cancer cases will rise from 14 million in 2012 to 22 million within the next two decades [1]. In particular, to date, breast cancer is the most common cancer in women [2,3]. The only species that has a comparable incidence of mammary tumors is the female dog [4]. Indeed, the estimate of cancer incidence in dogs ranges from 99.3 to 272.1 per 100,000 dog-years [5] and more than 40% of tumors in female dogs arise from the mammary gland [6–8]. It has been described that approximately 50% of canine mammary tumors (CMTs) are histologically malignant with a 20% rate of metastases [9]. This data suggests that it would be very relevant to identify rapid diagnostic and relevant prognostic markers in veterinary as in human medicine. In clinical practice, this may be achieved by the identification of biomarkers either from the tumor itself or the blood. The use of circulating biomarkers has advantages over the use of tissue biopsies due to their availability obtained by minimally invasive procedures, and the opportunity to withdraw numerous samples over several time points. Therefore, “liquid biopsy” is an excellent alternative for reflecting and providing information about the tumor status [10]. In recent years, measuring circulating cell-free DNA (cfDNA) in plasma has gained attention as a biomarker in some human tumors, such as pancreatic cancer [11], breast tumors [12,13], and rectal cancer [14,15]. Little, but promising, information is present in veterinary medicine [16–18]. Several hypotheses have been postulated about cfDNA origin: its presence in the bloodstream could be due to i) DNA leakage resulting from tumor necrosis or apoptosis, ii) lysis of circulating cancer cells or micrometastases, or iii) unrevealed mechanism of active and spontaneous release [19,20]. DNA released from necrotic cells is cleaved into fragments of variable length that can reach up to 10,000 base pair (bp), called long fragments, whereas apoptotic cells cleave their own DNA into shorter fragments reaching a maximum of 180-200bp, called short fragments [11,20,21]. In healthy individuals, the main source of cfDNA is thought to be apoptotic cells. In contrast, necrotic cell death is a frequent event in solid tumors, and DNA fragments released from the necrotic tumor cells are, then, more variable in length [20]. Also, necrosis and apoptosis can be variably regulated in cancer, and an increase in cfDNA has been observed in cancer-bearing human subjects [22]. More specifically, the presence of long DNA fragments in blood and a disarranged ratio between long and short/total fragments, known as integrity index, could reflect the presence of cancer in humans [12,14,15,22,23].

The aim of this study was to determine the amount of short and long cfDNA fragments and the cfDNA integrity index in mammary tumor-bearing dogs to assess its potential application as diagnostic/prognostic marker and to study its relation with tumor necrosis/apoptosis. The amount of necrosis and apoptosis in CMTs was assessed by histology and immunohistochemical analysis of anti-apoptotic (Bcl-2) and pro-apoptotic (Bax and Bad) proteins.

## Materials and Methods

### Subjects’ enrollment, histopathology, and ‘follow-up’ data

The 78 mammary nodules obtained from 44 female dogs (mean age at the initial evaluation was 9.8 years, range 5–15 years) included in this study were selected from the archive of the Diagnostic Service of Veterinary Anatomical Pathology (www.simbavet.org), Department of Comparative Biomedicine and Food Science, University of Padua, Italy. Submission to the service requires a privacy and informed consent form that allows research studies on the submitted material without other sampling on animals. The study, therefore, did not require additional ethical approval. The samples were not specifically collected for this study and they were submitted from veterinary clinical practitioners between 2010 and 2013. Breed, age at time of tumor diagnosis, and reproductive status of the subjects were recorded and number of histological visible neoplastic nodules (single/multiple), tumor diameter (post-fixation at histology), mitotic index (number of mitotic figures in 10 high power magnification fields [hpfs] in the most proliferative areas), and vascular invasion at the periphery of the tumor were assessed (Table 1). Samples of surgically resected CMTs and corresponding lymph nodes (when available) were fixed in 10% buffered formalin and then routinely processed for histopathology. Classifications of CMTs were performed by a board-certified veterinary pathologist (VZ), according to the updated classification system [24]. Grading and mitotic cell count of the tumors were performed according to Peña and co-authors [25]. After slide scanning, necrotic areas within the tumor were manually calculated using D-Sight software (A. Menarini diagnostic, Firenze, Italy) and expressed as percentage on total tumor area on 4-μm thick sections obtained from sagittal sectioning of each nodule.

**Table 1:** Signalment and morphological features of the canine mammary tumors included in the study.

ID subject	Breed	Age (years)	Reproductive status	Number of nodules per subject	Histopathological diagnosis	Gra-ding	Lesion size (cm)	Mitotic index	Benign (B) or malignant (M) lesion	Simple (S) or complex (C) lesion	Presence of emboli
1	Pointer	12	IF	2	Complex Carcinoma	II	1.5	4	MM	CC	NO
					Complex Carcinoma	II	0.7	4			NO
2	Miniature Pinscher	10	IF	1	Simple Tubular Carcinoma	II	0.7	4	M	S	NO
3	Mixed	12	IF	1	Simple Tubular Carcinoma	I	0.2	4	M	S	NO
4	Shih Tzu	9	IF	2	Complex Carcinoma	II	10	6	MM	CC	NO
					Complex Carcinoma	II	5	4			NO
5	Boxer	10	IF	3	Complex Carcinoma	I	0.6	8	BM	CC	NO
					Carcinoma in Benign Mixed Tumor	I	1	4			NO
					Complex Adenoma	n/a	0.4	2			NO
6	Dobermann Pinscher	7	IF	2	Complex Adenoma	n/a	1	0	BB	SC	NO
					Simple Adenoma	n/a	0.4	2			NO
7	Brittany	10	IF	1	Solid Carcinoma	I	0.3	4	M	S	NO
8	Dachshund	9	IF	2	Complex Adenoma	n/a	1	0	BB	CC	NO
					Benign Mixed Tumor	n/a	1.5	0			NO
9	German Shepherd Dog	10	IF	1	Simple Tubulopapillary Carcinoma	II	3	10	M	S	NO
10	Standard Poodle	9	IF	3	Simple Tubular Carcinoma	I	0.3	5	BM	SC	NO
					Complex Carcinoma	I	2	4			NO
					Simple Adenoma	n/a	0.4	0			NO
11	Mixed	11	IF	2	Complex Adenoma	n/a	1.8	1	BB	CC	NO
					Benign Mixed Tumor	n/a	0.7	0			NO
12	Maltese	12	IF	1	Micropapillary Carcinoma	III	5	10	M	S	YES
13	English Setter	5	NF	1	Simple Tubulopapillary Carcinoma	MET	3	10	M	S	NO
14	Mixed	12	IF	1	Complex Carcinoma	I	1	5	M	C	NO
15	Beagle	12	IF	2	Complex Carcinoma	II	0.7	4	BM	CC	NO
					Benign Mixed Tumor	n/a	0.4	0			NO
16	Maltese	10	IF	2	Complex Adenoma	n/a	0.7	0	BB	CC	NO
					Benign Mixed Tumor	n/a	0.2	0			NO
17	Mixed	8	IF	2	Carcinoma and Malignant Myoepithelioma	I	0.8	12	MM	SC	NO
					Simple Cystic-Papillary Carcinoma	I	1.3	5			NO
18	Airedale terrier	8	NF	4	Complex Carcinoma	I	1.3	6	BM	SC	NO
					Mixed Carcinoma	I	0.7	7			NO
					Complex Adenoma	n/a	1.1	0			NO
					Simple Adenoma	n/a	0.8	1			NO
19	German Shepherd Dog	10	NF	3	Anaplastic Carcinoma	MET	n/a	0	MM	SC	YES
					Simple Tubular Carcinoma	I	0.5	0			NO
					Complex Carcinoma	II	0.9	0			NO
20	Puli	12	IF	3	Complex Adenoma	n/a	0.5	0	BB	SC	NO
					Intraductal Papillary Adenoma	n/a	0.6	0			NO
					Ductal Adenoma	n/a	0.5	1			NO
21	Dobermann Pinscher	6	NF	2	Simple Tubular Carcinoma	I	0.7	2	BM	SS	NO
					Simple Adenoma	n/a	0.4	2			NO
22	Dachshund	10	IF	1	Complex Adenoma	n/a	1.5	0	B	C	NO
23	Wire Fox Terrier	9	IF	1	Complex Carcinoma	I	0.8	4	M	C	NO
24	Mixed	5	IF	1	Simple Tubular Carcinoma	I	0.4	3	M	S	NO
25	Dalmatian	13	IF	2	Carcinoma and Malignant Myoepithelioma	MET	0.8	4	MM	CC	NO
					Complex Carcinoma	I	0.4	4			NO
26	Mixed	15	IF	3	Carcinoma and Malignant Myoepithelioma	III	2	16	MM	SC	NO
					Simple Tubular Carcinoma	II	0.5	6			NO
					Complex Carcinoma	I	0.2	4			NO
27	German Shepherd Dog	8	IF	1	Simple Tubular Carcinoma	III	2	25	M	S	YES
28	Miniature Pinscher	13	IF	3	Intraductal Papillary Carcinoma	III	0.5	5	BM	SC	NO
					Complex Carcinoma	I	0.5	2			NO
					Complex Adenoma	n/a	0.5	0			NO
29	Bullmastiff	7	NF	1	Simple Cystic-Papillary Carcinoma	I	1	4	M	S	NO
30	Bulldog	10	IF	1	Carcinoma in Benign Mixed Tumor	I	0.8	4	M	C	NO
31	German Shepherd Dog	8	IF	3	Carcinoma and Malignant Myoepithelioma	I	0.7	5	MM	CC	NO
					Complex Carcinoma	III	0.6	12			NO
					Complex Carcinoma	I	0.3	3			NO
32	Mixed	10	NF	1	Complex Carcinoma	III	0.9	4	M	C	NO
33	Labrador Retriever	13	NF	1	Adenosquamous Carcinoma	MET	0.7	10	M	S	NO
34	Standard Poodle	7	NF	1	Simple Tubular Carcinoma	I	1.2	2	M	S	NO
35	German Shepherd Dog	12	NF	2	Simple Tubular Carcinoma	II	0.3	4	MM	SC	NO
					Complex Carcinoma	I	1.5	4			NO
36	Mixed	10	IF	1	Intraductal Papillary Carcinoma	II	1.4	11	M	S	NO
37	Golden Retriever	10	IF	1	Solid Carcinoma	III	1	1	M	S	YES
38	Mixed	7	IF	1	Intraductal Papillary Carcinoma	II	3	9	M	S	NO
39	Yorkshire Terrier	12	IF	3	Simple Tubulopapillary Carcinoma	I	0.4	3	BM	SC	NO
					Complex Adenoma	n/a	0.3	0			NO
					Benign Mixed Tumor	n/a	0.4	0			NO
40	Yorkshire Terrier	11	NF	3	Simple Tubular Carcinoma	III	0.6	25	MM	SC	NO
					Complex Carcinoma	I	1.2	0			NO
					Intraductal Papillary Carcinoma	I	1.4	0			NO
41	German Shepherd Dog	9	IF	1	Complex Adenoma	n/a	0.7	1	B	C	NO
42	Mixed	6	IF	2	Anaplastic Carcinoma	III	n/a	7	MM	SC	NO
					Complex Carcinoma	II	2.4	10			NO
43	German Shorthaired Pointer	10	IF	1	Benign Mixed Tumor	n/a	1.4	1	B	C	NO
44	Mixed	12	NF	2	Simple Tubular Carcinoma	MET	1	12	BM	SC	YES
					Benign Mixed Tumor	n/a	1.7	0			NO

IF, intact female; NF, neutered female; n/a, not applicable; MET, metastatic; M, presence of one malignant tumor; MM, presence of more than one malignant tumors; B, presence of one benign tumor; BB, presence of more than one benign tumors; BM, presence of benign and malignant tumors; S, presence of one simple tumor; SS, presence of more than one simple tumors; C, presence of one complex tumor; CC, presence of more than one complex tumors; SC, presence of simple and complex tumors.

At the beginning of the study (2013) and after one year, clinical data of the neoplastic subjects were obtained from referral veterinarians through regular telephone interviews to collect at least one-year post-diagnosis follow-up for each subject. Overall survival (OS) was calculated as the time from initial surgery to death. Death was considered related to the mammary tumor when an animal died naturally or was euthanized in presence of metastases identified by diagnostic imaging. Necropsy data were not available.

In the study, non-neoplastic subjects were also included. Animals were presented at The Veterinary Teaching Hospital of the University of Padua—OVUD—http://www.unipd.it/universita/sedi-strutture/ospedale-veterinario that has been approved by the European Association of Establishments of Veterinary Education—EAEVE. They were not specifically referred to clinicians for this project but they were presented for clinical assistance, for routine annual physical examination, or for routine sterilization. Since the beginning of the study (2013), data from the physical examination, complete blood cell count (CBC), and biochemical profile were obtained from the OVUD clinicians who registered them after clinical examinations.

After one year, control physical examinations on each of these subjects were performed and registered by OVUD clinicians in order to verify the general health status and to identify whether a mammary nodule or any other clinically detectable neoplasia or disease (in case of healthy subjects) were present. Owners were informed on the study but since no invasive procedures were performed on the subjects in addition to what required for routine assistance of diseased animals, no ethical approval was needed.

### Cell cultures

For protein extraction and western blot analysis, CF41 cells (canine mammary carcinoma cell line, ATCC CRL-6232) were cultured in Dulbecco’s Modified Eagle’s Medium (DMEM) containing 10% Fetal Bovine Serum (FBS; Gibco, Life Technologies), 1% (5000 U/ml) penicillin and 5 mg/ml streptomycin. The cells were maintained at 37°C in a humidified 5% CO_2_ atmosphere.

### Western blot analysis

For protein extraction, CF41 cells were mechanically harvested in 5 ml of ice-cold PBS and centrifuge at 800 x g for 4 minutes. After supernatant removal, cells were lysed with 1 ml of Lysis Buffer (150mM NaCl, 1.0% Triton X-100, 50 mM Tris, pH 8.0), incubated on ice for 30 minutes and centrifuged at 17000 x g for 15 minutes at 4°C. The extracted proteins were quantified using the BCA protein assay kit (Thermo Scientific, Loughborough, UK) and stored at -80°C.

For western blot analysis, proteins were resolved in 12% polyacrylamide gels, transferred onto nitrocellulose membranes, blocked with 3% non-fat dry milk in TBS-T buffer [50 mM Tris, 150 mM NaCl, 0.05% Tween 20 (Sigma Aldrich), pH 7.5] for 1 h, probed with the appropriate antibodies at 4°C overnight: Bcl-2 (N-19 sc-492, rabbit pAb, Santa Cruz Biotechnology, 1:1000 dilution), Bax (P-19, sc-526, rabbit pAb, Santa Cruz Biotechnology, 1:1000 dilution), and Bad (Y208, ab32445, rabbit mAb, Abcam, 1:1000 dilution). The membranes were washed in TBST and then incubated with corresponding anti-rabbit sheep secondary antibody (Dako, Glostrup, Germany, 1:4000 dilution) for 1 h at room temperature. After further washing in TBS-T, the blot was developed using an automatic film-processing unit (X-Ograph, Gloucestershire, UK).

### Immunohistochemistry

Immunohistochemistry (IHC) was performed to detect Bcl-2, Bax, and Bad expression in the 78 tumors. For Bcl-2 and Bad analysis, the sections (4μm) were processed with an automatic immunostainer (BenchMark XT, Ventana Medical Systems, Tucson, Arizona, USA). Briefly, the automated IHC protocol included high temperature antigen unmasking (30 min at 95°C) and incubation at room temperature of the primary antibody (44 min for Bcl-2, dilution 1:150 and 1 h for Bad, dilution 1:1200).

For Bax analysis an IHC manual protocol was performed. Briefly, IHC protocol included high temperature antigen unmasking (20 minutes at 95°C) in citrate buffer (pH 6.00), 10 minutes 2% hydrogen peroxide incubation, 10 minutes Protein Block incubation, 1 h incubation with Bax antibody at room temperature (dilution 1:100), 10 minutes of incubation with secondary antibody (Biotinylated Goat Anti-Rabbit, Abcam, Cambridge, UK), 5 minutes DAB chromogen incubation, 3 minutes hematoxylin counterstaining, dehydration and, finally, mounting medium application.

Sections of canine lymph nodes were used as positive controls for the three markers. In negative controls PBS buffer replaced the primary antibodies.

The semi-quantitative IHC evaluation of protein expression was determined as percentage (counting 100 cells per field in at least 10 fields at 40x) and intensity (scored from 0.5 to 3) of positive cells. Subsequently, two different scoring systems were also applied:

- IRS scale (0–12 points): product of the score given to the percentage of positive cells (negative → 0; positivity in 1% to 10% of cells → 1; 11% to 50% → 2; 51% to 80% → 3; and 81% to 100% → 4) and the score given to the intensity (from 0.5 to 3).
- Allred score (0–8 points): sum of the score given to the percentage of positive cells (negative → 0; positivity in <1% of cells → 1; 1% to 10% → 2; 10% to 33% → 3; 34% to 66% → 4; and 67% to 100% → 5) and the score given to the intensity (from 0.5 to 3).

### Plasma samples collections and cfDNA purification

Blood samples from neoplastic subjects (n = 44) had been collected and stocked from clinical practitioners at time of surgical excision of mammary nodule/s. This procedure had been established since 2010 involving clinical practitioners into research collaboration with our Anatomical Pathology Unit. The collaboration included submission of surgical excised tumors to our Diagnostic Service (www.simbavet.org) for histology and preservation of an aliquot of pre-surgical blood samples (2mL). This is a procedure that has been established for any case of tumor (not only mammary) that was submitted to our Diagnostic Service and was not specifically performed for this study. Similarly, blood samples (2mL) from non-neoplastic subjects (n = 32) were also included. This sampling was performed for routine analysis or pre-sterilization from OVUD clinicians. No additional sampling/procedures were carried out on the subjects, since surgical excision and blood sampling were performed as primary treatment and diagnostic procedures. Animal owners signed an informed consent for blood sampling and surgery as for routine clinical practice. No additional informed consents or ethical approval were required.

For stocking, 2mL of peripheral blood were drawn into a blood collection tube (containing EDTA additive) from clinicians and practitioners and plasma was immediately separated by centrifugation at 3000 x g for 10 minutes, and stored at -20°C, until further analysis [15,21,23].

After collection of samples from the clinics, the cfDNA was purified in our lab from 500 μl of plasma with the QIAamp UltraSens Virus Kit (Qiagen, Hilden, Germany) according to the manufacturer’s instructions, without additional centrifugation, as previously described [15,21,23]. The cfDNA preparations were eluted in 60 μl of elution buffer and stored at -20°C.

### Quantitative PCR of plasma cfDNA fragments

Quantitative real-time PCR (qPCR) was used to amplify and quantify the short and long cfDNA fragments. LINE-1 gene sequence (GenBank accession number: AY266086.1), which is the most abundant repeat sequence present in canine genome [26], was used as a target of the qPCR in order to maximize the sensitivity of cfDNA quantification. A common reverse primer and two different forward primers were used to amplify on the same region the cfDNA fragments with different length as described below. The first forward primer pair amplified a short fragment of 99bp (LINE-99) that was designed inside the long fragment of 218bp (LINE-218) amplified by the second forward primer pair (S1 Fig).

The quantification obtained using the LINE-99 primer pair represented therefore the total circulating cfDNA (ng/ml of plasma) (herein referred as “short fragments”), whereas the LINE-218 primer pair identifies long cfDNA fragments because these fragments can include more template of LINE sequence. These longer fragments (herein referred as “long fragments”) are presumably released from non-apoptotic cell processes. The ratio between long and short fragments showed the integrity index of cfDNA in each plasma sample.

The qPCR reaction was performed using 7300 Real-Time PCR System (Applied Biosystem, Milan, Italy). Briefly, the reaction was performed in 20 μL reaction volumes containing 2X FluoCycle II Master Mix for Probe (EuroClone, Milan, Italy), 0.9 μM of each primer, 0.25 μM of probe (5’ 6-FAM and 3’ TAMRA labeled) and 1 μL of sample. The real-time PCR conditions consisted of an initial denaturation step for 5 minutes at 95°C followed by 40 cycles of denaturation for 15 seconds at 95°C and annealing/extension for 1 minute at 60°C.

A negative control (without the template) was performed in each plate and the samples were analyzed in triplicate. The qPCR plate design was performed in a blinded fashion without knowledge of the specimen identity.

The absolute amount of cfDNA fragments in each sample was determined by the standard curve with the 6-log linear range using 10-fold dilutions (from 10 ng to 10 fg) of genomic DNA from peripheral lymphocytes of a healthy subject as previously reported [26].

The standard curve presented in each sample plate showed the average value and standard deviation of the percentage reaction efficiency, curve slope and R square as 100.891 ± 2.093, -3.302 ± 0.049 and 0.998 ± 0.001 for the LINE-99 and 103.5 ± 5.844, -3.245 ± 0.128 and 0.997 ± 0.001 for the LINE-218, respectively.

### Statistical analysis

To verify mean differences among groups, the Student’s t test in case of two samples groups and the one-way ANOVA in case of more than two samples groups were used. Tukey correction has been used for the multi-comparison issue. The Pearson correlation test was applied to analyze associations between variables. A multivariate logistic regression model with an automatic variable selection strategy (based on AIC criteria) was used to identify the best discriminative model for neoplastic and non-neoplastic and for benign and malignant categories. Leave on out cross-validation strategy was applied to estimate average specificity and sensitivity of the model. Kaplan-Meier curves were applied for survival analyses. Values were applied after logarithmic transformation. Level of significance was fixed as P<0.05.

## Results

### Clinical features and histopathology of CMTs

The 32 non-neoplastic subjects did not present mammary nodules or other tumors at clinical examination both at the beginning of the study and after one year. The non-neoplastic diseased subjects (n = 17) manifested variable pathological processes and CBC and/or biochemistry alterations. Both inflammatory and non-inflammatory diseases were randomly included to avoid any type of bias (S1 Table). Among the (n = 15) healthy subjects 2 animals manifested significant CBC and/or biochemistry alterations with pronounced leukocytosis and were moved to the diseased group (S1 Table). Three healthy subjects presented slightly altered CBC and were therefore removed. The total of non-neoplastic subjects was therefore 19 and 10, for diseased and healthy dogs, respectively.

Histology of the samples from the 44 neoplastic subjects showed 54 malignant and 24 benign tumors of simple (n = 33) and complex subtypes (n = 45) (Table 1). Twenty-seven malignant tumors were grade I (9/27 with necrosis), 13 grade II (6/13 with necrosis), and 9 grade III (7/9 with necrosis). Positive metastatic lymph nodes were present in 5/17 cases, and 3/5 primary metastatic tumors showed necrotic areas. Five grade III carcinomas showed peripheral vascular invasion, and 2/5 had lymph node metastases. Necrosis in the 25/54 carcinomas ranged from 0.6% to 31.9% of the tumor (av. 9.1%). Eighteen dogs presented single malignant tumors, three dogs single benign tumors, and 23 dogs presented multiple, benign or malignant (or both) tumors (Table 1). The 78 studied CMTs were classified as reported in Table 1: they were mainly complex carcinomas (n = 20) and adenoma (n = 11), simple tubular/tubulopapillary/cystic-papillary carcinomas (n = 17), and benign mixed tumors (n = 7). The vast majority of mammary lesions was surrounded by healthy or hyperplastic tissue. Considering the heterogeneity of canine mammary tumors, no further selection of samples was performed in the study to test the significance of the cfDNA markers in the spontaneous conditions.

### Western blot analysis

The immunoblotting experiments performed on the protein extract from the CF41 cells recognized the 26 KDa, 23 KDa, and 23 KDa proteins, respectively corresponding to the expected molecular mass of Bcl-2, Bax and Bad proteins.

### Immunohistochemistry

In the canine mammary gland samples, Bcl-2, Bax, and Bad staining was diffusely cytoplasmic and it was observed in both tumor and healthy/hyperplastic tissues and in both epithelial/luminal and myoepithelial/basal cells. In tumors composed mainly by the proliferation of a single epithelial/luminal population, only its positivity was counted, whereas in biphasic tumors with proliferation of both epithelial/luminal and myoepithelial/basal cells (such as complex tumors, benign mixed tumors, carcinoma-mixed type, and carcinoma-and-malignant myoepithelioma) the positivity was counted separately for the two populations. Lymph nodes sections showed expected positivity for all three markers. Cutaneous adnexa, especially the apocrine glands, were also used as positive internal control because of their strong positivity. Specifically, Bcl-2 was highly expressed in the basal cells of the glandular adnexa rather than in the luminal cells, whereas Bax and Bad positivity was higher in the luminal cells than in the basal cells.

**Fig 1:**
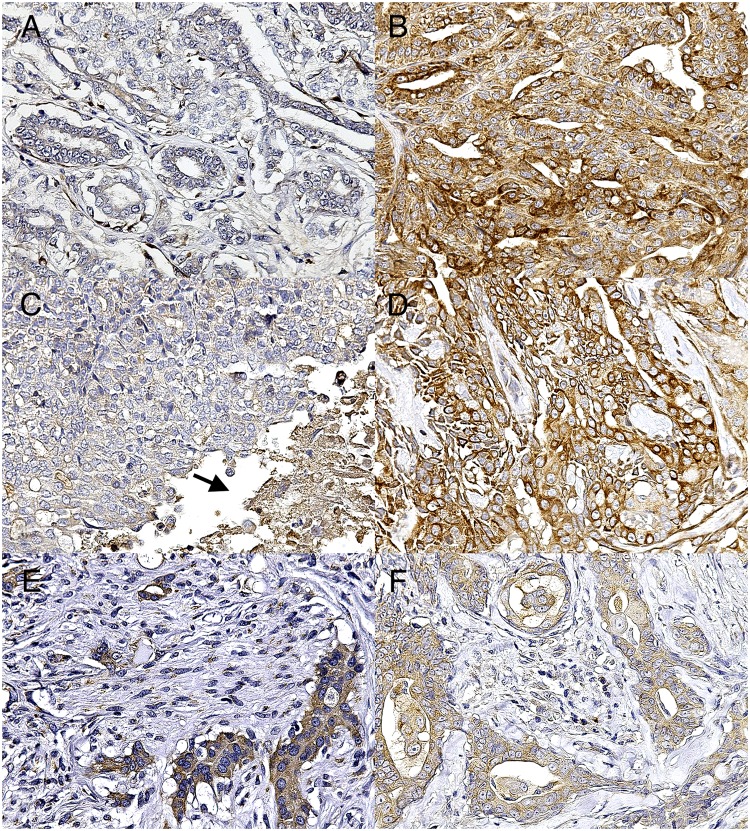
Photomicrographs of IHC in canine mammary gland. 1A-1F. IHC for Bcl-2, Bax, Bad. (A) Complex adenoma. Weak expression of Bcl-2 in benign tumors. (B) Simple tubulopapillary carcinoma, grade II. Strong expression of Bcl-2 in malignant tumors. (C) Intraductal papillary carcinoma, grade II. Weak expression of Bcl-2 in tumors with necrotic areas (arrow). (D) Complex carcinoma, grade II. Strong expression of Bcl-2 in tumors without necrosis. (E) Complex carcinoma, grade II. Higher expression of Bax in epithelial cells than in myoepithelial cells. (F) Complex carcinoma, grade I. Higher expression of Bad in epithelial cells than in myoepithelial cells.

Considering tumor lesions (78) and apoptotic markers expression (Table 2 and S2 Table), Bcl-2 was more expressed in malignant tumors (P<0.01 for positive cells intensity and IRS scale) than in benign tumors (Fig 1A and 1B) and in healthy tissue. Bcl-2 expression was similarly augmented in metastatic, necrotic, and non-necrotic lesions, however a higher increase, significant for the two scoring systems, was observed in non-necrotic tumors indicating that blocking of apoptosis is more evident in tumors without necrosis (Fig 1C and 1D). Bax and Bad were only slightly increased in malignancies and this trend was particularly evident, despite not significant, in necrotic tumors (Table 2). Additionally, Bax (IRS scale, r = 0.5, and Allred score, r = 0.6; P<0.05) and Bad (luminal %, r = 0.5, P = 0.06) expression in luminal cells, indicative of a slight increase of apoptosis, positively correlated with percentage of tumor necrosis.

**Table 2:** Immunohistochemistry results (mean ± standard deviation) of Bcl-2, Bax, and Bad expression in luminal epithelial cells of canine mammary tumors.

	Bcl2				Bax				Bad			
	Positive cells %	Positive cells intensity	IRS	Allred	Positive cells %	Positive cells intensity	IRS	Allred	Positive cells %	Positive cells intensity	IRS	Allred
Healthy/hyperplastic tissue	100.00 ± 0.00	0.96 ± 0.31	3.85 ± 1.23	5.96 ± 0.31	91.43 ± 19.57	0.61 ± 0.21	2.30 ± 0.96	5.48 ± 0.44	100.00 ± 0.00	0.59 ± 0.24	2.36 ± 0.95	5.59 ± 0.24
Benign tumors (24)	90.50 ± 27.62	0.95 ± 0.43	3.65 ± 1.92	5.65 ± 1.15	90.50 ± 19.32	1.08 ± 0.47	3.93 ± 1.79	5.98 ± 0.57	97.50 ± 7.86	1.03 ± 0.47	4.05 ± 1.90	6.03 ± 0.47
Malignant tumors (54)	**95.81 ± 16.65**	1.35 ± 0.57**	5.27 ± 2.14**	**6.21 ± 1.12**^MS^	86.44 ± 23.95	1.30 ± 0.54	4.69 ± 2.23	6.06 ± 0.79	99.35 ± 3.27	1.10 ± 0.48	4.38 ± 1.93	6.10 ± 0.48
Metastatic tumors (5)	92.00 ± 10.95	1.70 ± 0.76	5.90 ± 1.95	6.70 ± 0.76	100.00 ± 0.00	1.13 ± 0.63	4.50 ± 2.52	6.13 ± 0.63	96.00 ± 8.94	0.80 ± 0.45	3.10 ± 1.88	5.80 ± 0.45
Necrotic malignant tumors (25)	93.04 ± 22.25	1.17 ± 0.49	4.59 ± 1.97	5.91 ± 1.38	**91.74 ± 17.23**	**1.35 ± 0.55**	**4.96 ± 2.17**	**6.22 ± 0.69**	**98.70 ± 4.58**	**1.15 ± 0.65**	**4.59 ± 2.61**	**6.15 ± 0.65**
Non-necrotic malignant tumors (29)	**99.00 ± 4.47**	**1.55 ± 0.60**	6.05 ± 2.11**	6.55 ± 0.60**	80.91 ± 28.77	1.25 ± 0.53	4.41 ± 2.32	5.89 ± 0.87	100.00 ± 0.00	1.04 ± 0.21	4.17 ± 0.83	6.04 ± 0.21

Most relevant results are shown in bold.

** significantly (P<0.01) different from benign tumors.

^MS^ marginally significantly (0.1<P<0.05) different from benign tumors.

In addition, tumor size was positively correlated with Bcl-2 expression in luminal cells (r = 0.4; P<0.0001) and marginal significance was found for a negative correlation with Bax IRS scale in luminal cells (r = -0.2; P = 0.05).

Within the tumor tissue, the IHC expression of Bcl-2, Bax, and Bad in luminal cells was positively correlated with their expression in myoepithelial cells (r = 0.7; r = 0.4; r = 0.2; P<0.01), but the three markers were generally more expressed in the luminal cells than in the myoepithelium (P<0.05) (Fig 1E and 1F). Grading, tumor subtype, and other tumor or subject-related features were not significantly correlated with markers expression.

### Circulating cfDNA in plasma

**Fig 2:**
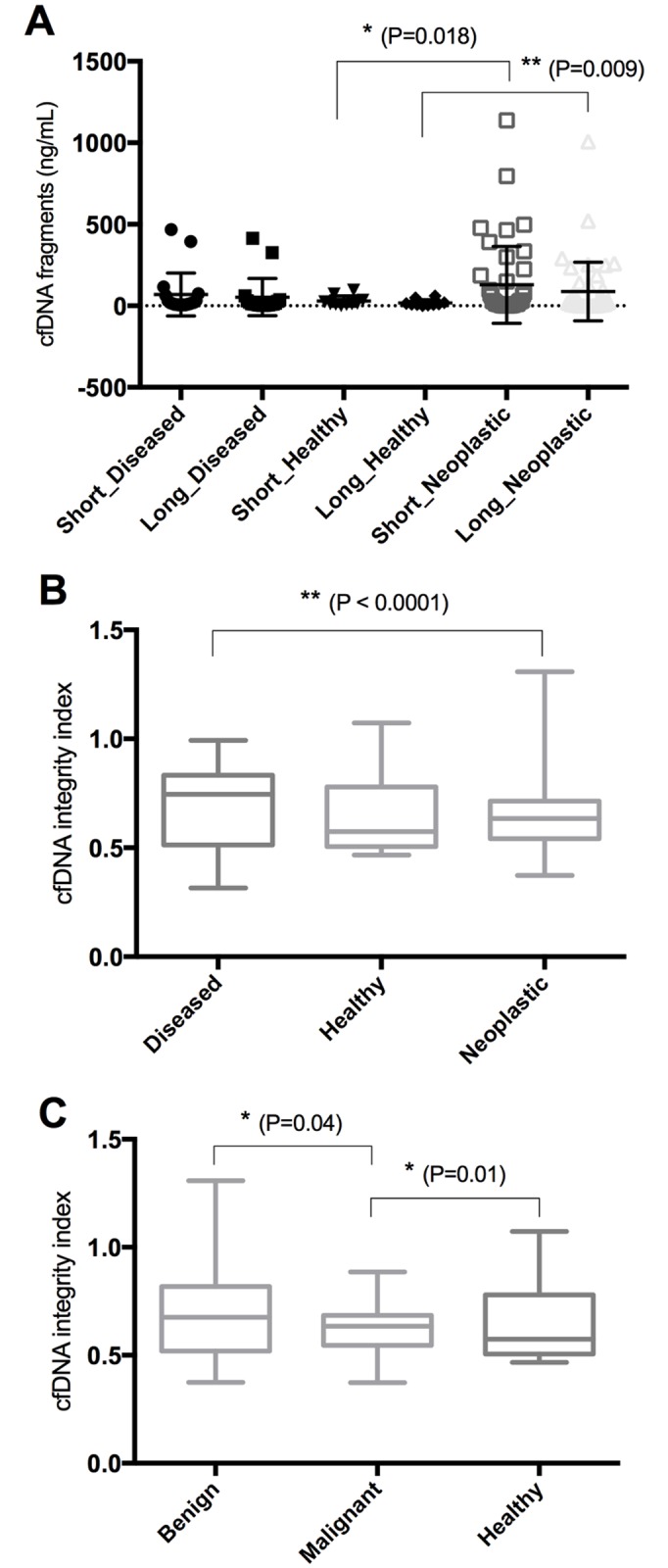
Circulating cell-free (cf) DNA concentrations in healthy dogs and dogs with neoplastic and non-neoplastic diseases (diseased). (A) Scatterplot of cfDNA short and long fragments. (B) Box and Whiskers of cfDNA integrity index (long/short fragments) in healthy dogs and dogs with neoplastic and non-neoplastic diseases (diseased). (C) Box and Whiskers of cfDNA integrity index in neoplastic dogs with benign or malignant tumors and in healthy dogs. Error bars represent standard deviation. *P<0.05; ** P<0.01.

Circulating cfDNA in plasma of dogs with neoplastic and non-neoplastic lesions was assessed for short (total) and long (non-apoptotic) cfDNA fragments concentration and then the integrity index was calculated (long/short). When analyzing cfDNA values from neoplastic subjects a significant outlier was found. This was a subject (n.42) carrying an anaplastic carcinoma with diffuse necrosis (16%) and massive vascular invasion, presenting short fragments = 1137 ng/ml, long = 1007,8 ng/ml, and integrity index = 0.89 (S1 Table). However, this subject was still included in the further analysis. Both healthy and non-neoplastic diseased dogs (n = 29) showed cfDNA values significantly different from neoplastic dogs (n = 44) (Fig 2). Healthy individuals had short (mean 28.8 ng/ml; range 6.5–51.1, 95% CI) and long (mean 18.25 ng/ml; range 3.9–32.6, 95% CI) fragments significantly lower than neoplastic subjects. The latter had short fragments ranging from 56.85 to 200.2 ng/ml in the central 95% interval (mean 128.5 ng/ml) and long fragments included between 33 and 142 ng/ml (95% CI, mean 87.48 ng/mL). The means for non-neoplastic diseased animals were 69 ng/ml (range 5.7–132.4 ng/mL) and 53.22 ng/ml (range 1.5–107.9 ng/ml) for short and long fragments, respectively. Within diseased individuals, two non-statistical “outliers” were found (Fig 2) that were respectively carrying an arrhythmogenic cardiomyopathy and signs of liver damage subsequently identified as cholangiohepatitis. The integrity index of neoplastic subjects (0.64, range 0.6–0.69) was lower than diseased animals (0.7, range 0.62–0.8) (P<0.0001) and than healthy individuals (0.65, range 0.51–0.78) (P>0.05) (Fig 2). Moreover, subjects with at least one malignant tumor showed a significantly lower (P<0.05) and less variable integrity index (0.64; range 0.60–0.68) than those with exclusively benign lesions (0.71; range 0.47–0.95) or healthy individuals (0.65, range 0.51–0.78). Subjects with necrotic lesions manifested a higher amount of short (mean: 172.7 ng/ml) and long cfDNA fragments (129 ng/ml) and a higher integrity index (0.66) than the subjects with non-necrotic lesions (short 108.6 ng/ml; long 60.5 ng/ml; integrity index 0.61) (P>0.05) (S2 Fig).

In order to test a correlation between cfDNA and apoptotic markers or other features, only subjects carrying a single lesion were included in the analyses. Specifically, the cfDNA integrity index negatively and positively marginally correlated with intensity of Bax+ luminal cells (r = -0.5; P = 0.06) and tumor size (r = 0.6; P = 0.05), respectively. Other tumor or subject-related features were not significantly correlated with cfDNA values.

In the multivariate logistic regression analysis, the best discriminative model for neoplastic and non-neoplastic subjects included only the integrity index variable. Looking at the ROC curves, this model was characterized by a modest predictive power (sensitivity and specificity respectively 61% and 75%). When assessing benign versus malignant lesions all the variables were maintained in the model reaching a higher power of discrimination, with sensitivity and specificity respectively of 72% and 75%.

Regarding survival analyses, cfDNA values were not significantly predictive for the clinical outcome.

## Discussion

Circulating cfDNA is extracellular DNA found in serum or plasma that can be purified, quantified, and eventually specifically amplified by polymerase chain reaction (PCR) [27]. The increase of cfDNA has been reported in various inflammatory diseases, and it has been found also in human patients with various types of cancer [28]. To date, circulating cfDNA in blood has been considered a promising biomarker in some human tumors and, today, cfDNA fragmentation represented by the integrity index (ratio between long and short cfDNA fragments) is studied for its ability to discriminate cancer patients from healthy controls [29]. Very few data are available for the canine species [16–18].

In this study, we quantified the amount of short and long cfDNA fragments by qPCR of the two amplicons with different lengths (LINE-99 and LINE-218), in plasma of CMTs-carrying and non-neoplastic dogs. To correlate cfDNA fragmentation with the events of necrosis and apoptosis [20,21,30], we performed IHC analysis of the Bcl-2 anti-apoptotic protein and the Bax and Bad pro-apoptotic proteins, and we precisely measured the percentage of the necrotic tumor area in each mammary sample.

We firstly tested and confirmed the specificity of the antibodies used for IHC analysis against canine Bcl-2, Bax, and Bad proteins obtained from canine CF41 cells lysate. This is a critical step when testing antibodies produced against human antigens in a different species [31]. Considering their expression in our cohort of patients, Bcl-2 was more expressed in malignant tumors than in healthy tissue and in benign tumors, as already reported in CMTs [32,33] and in several human tumors [34–37]. Evasion of apoptosis is one of the common mechanisms by which neoplastic clones can expand into tumor masses and metastasize [38]. Bcl-2 expression was effectively positively correlated also to tumor size. A slight non-significant increase of Bax and Bad pro-apoptotic proteins was also evident in malignant tumors. This feature supports the hypothesis that the tumor bulk is composed of a heterogeneous cells population in which the apoptotic process could be differently activated [39]. In addition, some higher values of expression of Bax and Bad and a lower expression of Bcl-2 were particularly evident into the necrotic lesions; this might account for the increased Bax and Bad expression registered in malignancies and might confirm that the two major mechanisms of cell death, apoptosis and necrosis, are simultaneously activated within the tumor damaged area [40,41]. Bax luminal IRS scale and Allred score and Bad+ luminal % significantly correlated with percentage of tumor necrosis. This study confirmed that when evaluating positive expression by IHC, percentage and intensity of positive cells and combining scoring systems (*i*.*e*. IRS scale and Allred score) should be applied, as already discussed within the literature [42].

In this study, we detected the amount of cfDNA fragments using a quantitative PCR analysis against a LINE-1 sequence. The use of repetitive DNA elements with a high copy number distributed throughout the genomic DNA can ensure a good sensitivity and accurate data also for those samples with very low quantity and degraded cfDNA [10]. Particularly, amplification of ALU repeats (ALU-qPCR 247/115) to detect long (247bp) and short (115bp) DNA fragments and cfDNA integrity index is now used in human cancer diagnostic and prognostic studies [23, 43–47].

In agreement with previously published data mainly in humans, in our work, the neoplastic subjects showed a higher amount of both short and long cfDNA fragments than the non-neoplastic diseased and healthy controls [11–15, 23, 43–47]. Only one study [16] compared circulating DNA in non-neoplastic with neoplastic dogs (lymphoma and other cancers) and found similar results. In our study, healthy subjects had always less than 100 ng/ml short and 60 ng/ml long fragments, however these cut off values were not specific. Moreover, the subjects with at least one malignant tumor also presented a higher amount of both short and long cfDNA fragments compared to benign lesions. The cfDNA integrity index, unlike other studies [11,12,14,15], was significantly lower in the neoplastic subjects than in the non-neoplastic diseased subjects and in malignant tumors compared to benign and healthy subjects. Recently, Madhavan and co-authors obtained data similar to ours in a study of human breast cancer [10]. They observed an increase of the amount of cfDNA fragments and a decrease of the integrity index in cancer patients rather than in healthy controls [10]. They suggested that a higher DNA fragmentation—and therefore an increase of short fragments—might be present in apoptotic cancer cells compared to apoptotic non-cancerous cells. This would imply that the extent of DNA fragmentation during apoptosis might be different between cancer and normal cells as also supported by the work of Giacona and co-authors [11]. They demonstrated that cfDNA from apoptotic cells of healthy individuals had from three- to five-fold multiples of nucleosome associated DNA length (identified as long fragments), and considerably longer fragments than pancreatic cancer patients [11]. In a more simplistic vision, it seems that the rate of evasion of apoptosis taking place in the studied tumors was not sufficient to determine an increased ratio, since both short and long fragments were augmented. In our samples, instead, the presence of necrosis was able to significantly increase the integrity index causing more long fragments to circulate within the plasma, in accordance with other works [20,21,23,30,43–47] and further supporting the necrotic origin of the fragments amplified by the LINE-218 primer pairs.

Individual variability of cfDNA values was generally very high, as previously reported both in dogs [16,18] and humans [48–50], particularly in necrotic tumors.

Regarding prognosis, a significant role of cfDNA was not demonstrated, but this might be due to too much variability in tumor subtype, grade, and size, all of which significantly affect prognosis [51]. Further studies should be completed to better test a prognostic role and to verify applicability of thresholds of cfDNA values.

Dogs with non-neoplastic diseases presented, in our study, a slightly higher amount of cfDNA fragments than the healthy dogs but still lower than neoplastic subjects. This could be due to the presence of a pathological process in the diseased subjects with necrosis and apoptosis as a source of cfDNA. However, variability of cfDNA levels in these subjects, possibly due to type and severity of diseases selected in the group and the resulting variations in the ratio of cell damage and turnover [52–54] is not surprising and prevents us from forming any robust conclusions.

Healthy dogs manifested amounts of cfDNA fragments comparable to human controls included in published papers [48–50] and to that reported in one study of the dog [16].

A major concern is to establish the cancerous origin of the quantified cfDNA into the bloodstream of neoplastic subjects and to be able to use it for diagnosis and prognosis but also for monitoring systemic therapies and detecting therapeutic targets and resistance mechanisms in each patient. This approach is widely studied in humans [55–58] and together with the analysis of circulating tumor cells (CTCs) it has recently received enormous attention for its potential clinical implication [55–56]. When comparing CTCs and cfDNA they both present advantages and disadvantages and could hypothetically be complementary [44,55]. Therefore, there has been an explosion of technologies for the quantification and analysis of both these markers from the non-invasive and highly accessible “liquid biopsies”, however there is still a lack of standardization [55,56,59]. Quantification and integrity index of cfDNA are still proved to be valid diagnostic and prognostic markers in human cancer [23,43–47,60]. However, enormous improvement in sensitivity and specificity of downstream analysis has been achieved for example by next generation sequencing (NGS), digital droplet PCR (ddPCR), and beads, emulsion, amplification, magnetics (BEAMing) PCR for tumor association, mutational analysis, and patient monitoring [55,61]. In this regard the high sensitivity and low costs of the PCR-based methods would make them very advantageous but still limited by their need of known and previously identified/characterized mutational targets. The heterogeneity both between and within tumor types and individual patients is a well-known concept [62] and because of this the NGS approach would allow a more comprehensive snapshot of the tumor genome however with much higher costs and longer processing time [55,56]. In dogs, different gene mutations and gene copy number aberrations have been identified in canine mammary carcinomas and in one study also in the circulating DNA of the same cancer-bearing dogs [17,63–65]. We strongly believe that this approach based on wide sequencing methodology or high sensitivity PCR-based technology would further implement our results. On the base of these preliminary results is now possible to further investigate tumor-associated genetic changes of the circulating nucleic acids and their prognostic role. In addition, also monitoring the post-surgical trend of cfDNA levels in the plasma could allow even more robust association with the tumor lesions as well as offer prognostic information. However, ethical considerations with regard to performing additional blood sampling and exams may limit this approach.

In terms of prognosis we could not find any significant correlation with survival and the tested parameters. Conversely, one study targeted SINE sequences into the bloodstream of 28 neoplastic subjects and detected a prognostic significance [18]. Further prospective analysis with robust follow-up data should be prompted to better elucidate this aspect.

The results of this work still seem promising. They show that cfDNA fragments (short and long) and integrity index allow identification of malignant and necrotic CMTs and might therefore be further studied as potential diagnostic and prognostic tests also in veterinary medicine. In addition, it seems that both apoptosis and necrosis cause an increase of circulating DNA with necrosis as major source of long fragments and related increase of integrity index as already demonstrated in humans [23,43–47]. This is the first study of cfDNA quantification and cfDNA integrity index evaluation in dogs carrying CMTs, and its results suggest that further studies should be designed to strengthen the diagnostic/prognostic role of cfDNA levels and integrity index, including the establishment of verified cut-off values associated with different diagnoses and prognoses.

## Supporting Information

S1 Fig Primer pairs and probe used to amplify long and short fragments of the canine LINE-1 retrotransposon sequence. (PNG)

S2 Fig (A) Scatterplot of cfDNA short and long fragments in subjects carrying either benign or malignant mammary tumors and either necrotic or non-necrotic mammary carcinomas; (B) Box and Whiskers of cfDNA integrity index (long/short fragments) in subjects carrying either necrotic or non-necrotic mammary carcinomas. (TIFF)

S1 Table Short and long DNA fragments and (integrity) index (long/short) quantified (ng/mL) by qRT-PCR in the plasma of non-neoplastic diseased, healthy, and mammary tumor-bearing (neoplastic) subjects. In brackets the number of subjects is indicated. For non-neoplastic diseased subjects a list of the diagnosed diseases is provided, the term inflammatory indicates an increased white blood cells count in the subject.
(XLSX)

S2 Table Signalment, diagnosis, tumor diameter, extent of tumor necrosis, and immunohistochemical expression of Bcl-2, Bax, and Bad proteins for the dogs included in the study. (XLSX)
