# Supplementary figures and images for: Circulating Cell-Free DNA in Dogs with Mammary Tumors: Short and Long Fragments and Integrity Index
Source: PLoS One. 2017 Jan 12;12(1):e0169454. doi: 10.1371/journal.pone.0169454 (PMC5231265; doi:10.1371/journal.pone.0169454)

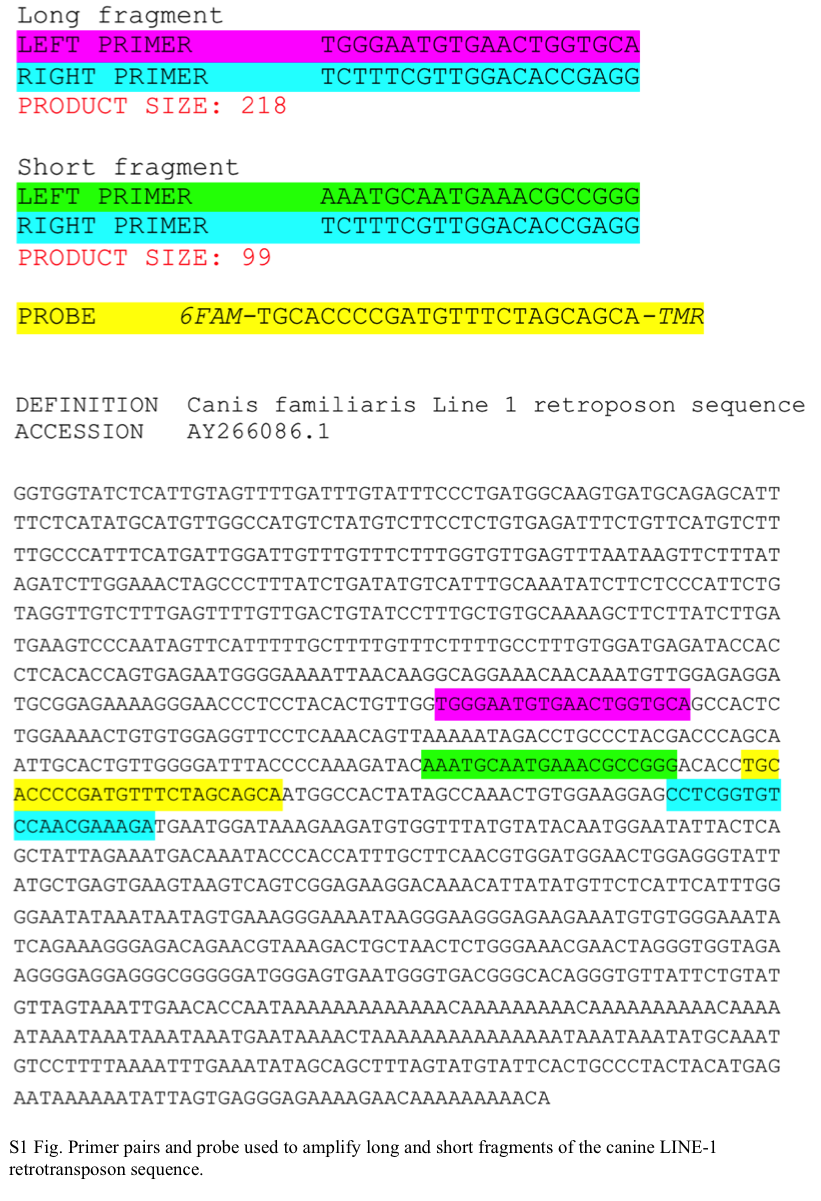

Supplement: S1 Fig — (PNG) [file pone.0169454.s001.png]

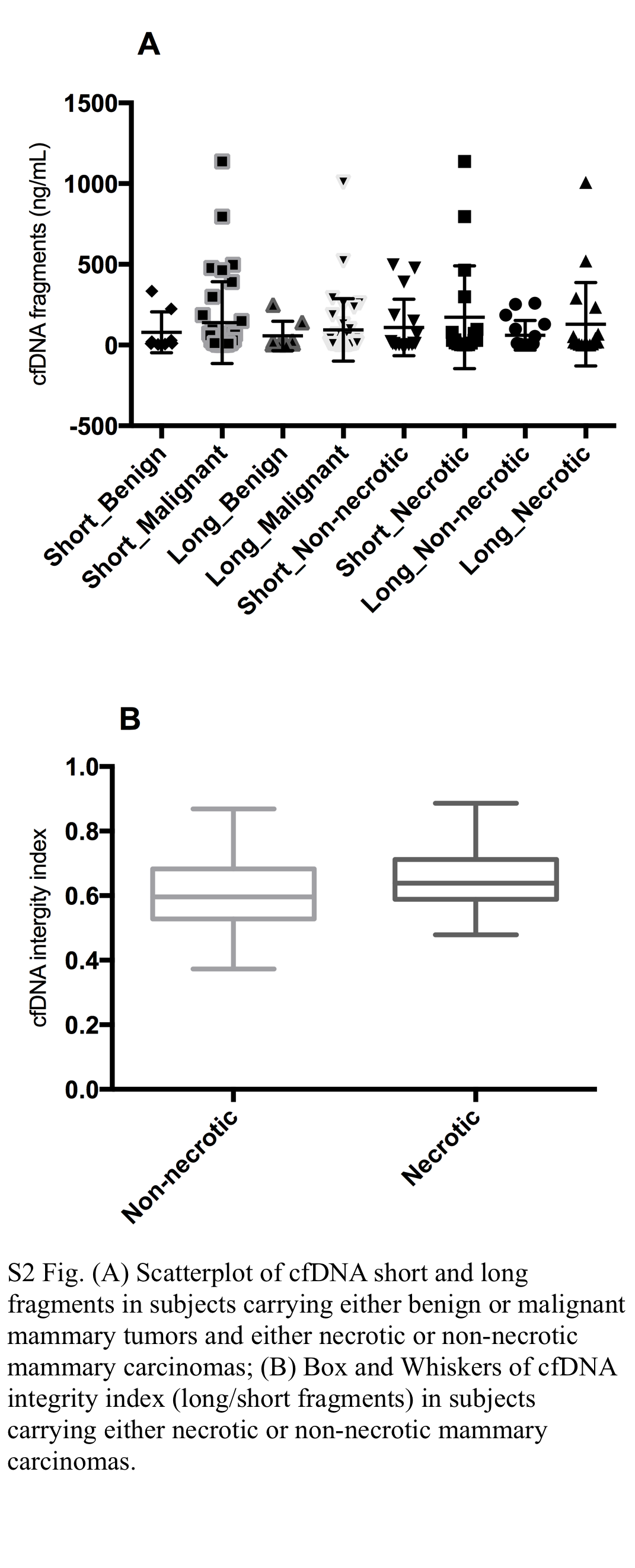

Supplement: S2 Fig — (TIFF) [file pone.0169454.s002.tiff]
